# Supplementary figures and images for: C5aR1 antagonism alters microglial polarization and mitigates disease progression in a mouse model of Alzheimer’s disease
Source: Acta Neuropathol Commun. 2022 Aug 17;10:116. doi: 10.1186/s40478-022-01416-6 (PMC9386996; doi:10.1186/s40478-022-01416-6)

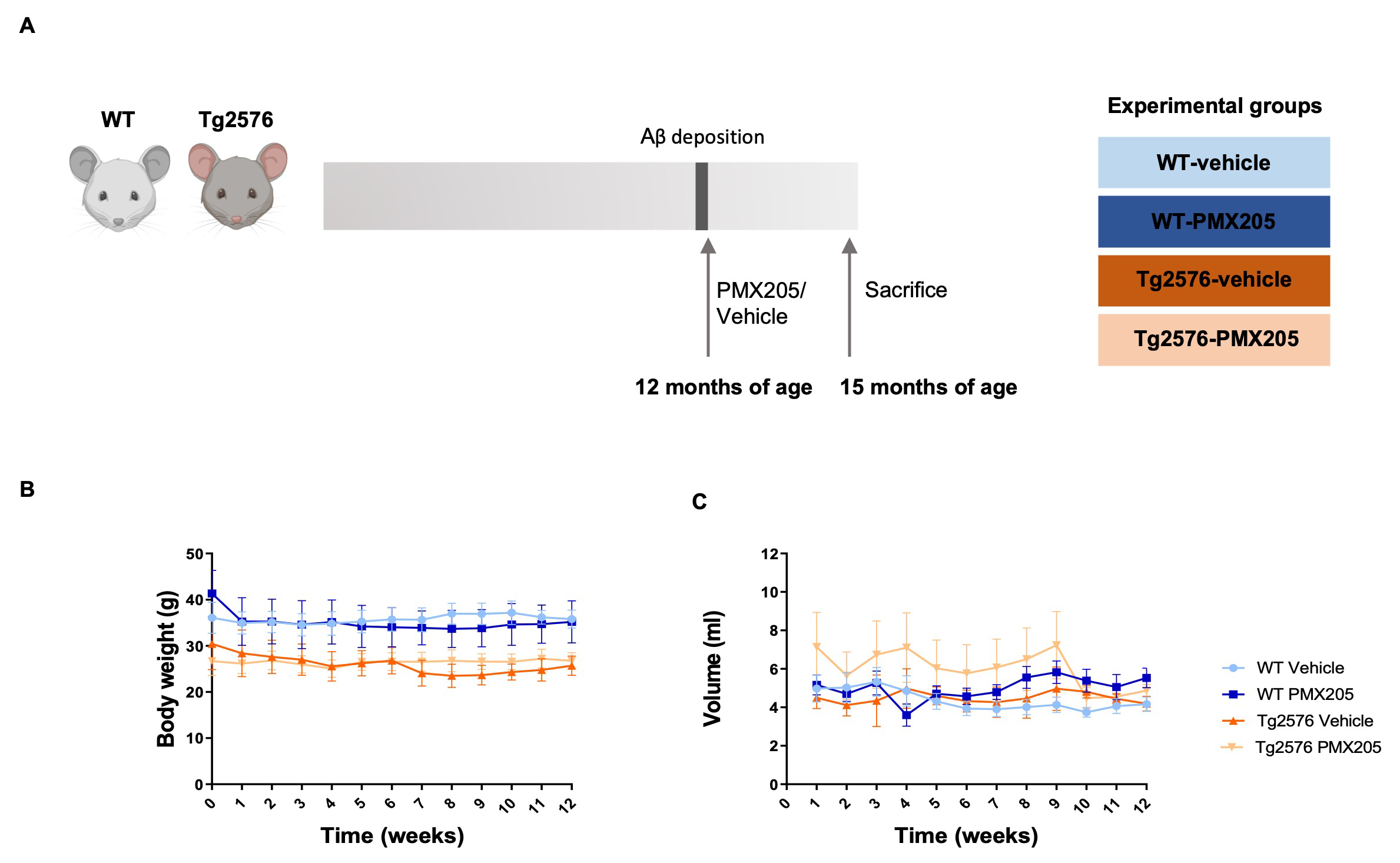

Supplement: Supplementary file 1 — Additional file 1: Figure S1. PMX205 treatment of Tg2576 and WT mice. (A) Schematic diagram of experimental design showing that Tg2576 mice and WT littermates were treated or not with 20 µg/ml of PMX205 for 12 weeks from 12-15 months of age. (B-C). During the duration of the treatment, mice body weight was recorded every week and no significant changes were observed in any of the experimental groups. Average drinking volume/week for each mouse showed no significant changes between the PMX205 and the control groups (C). Data shown as Mean ± SEM. Statistical analysis used a two-way ANOVA followed by Tukey’s post hoc test, n = 4-5 per group. [file 40478_2022_1416_MOESM1_ESM.tif]

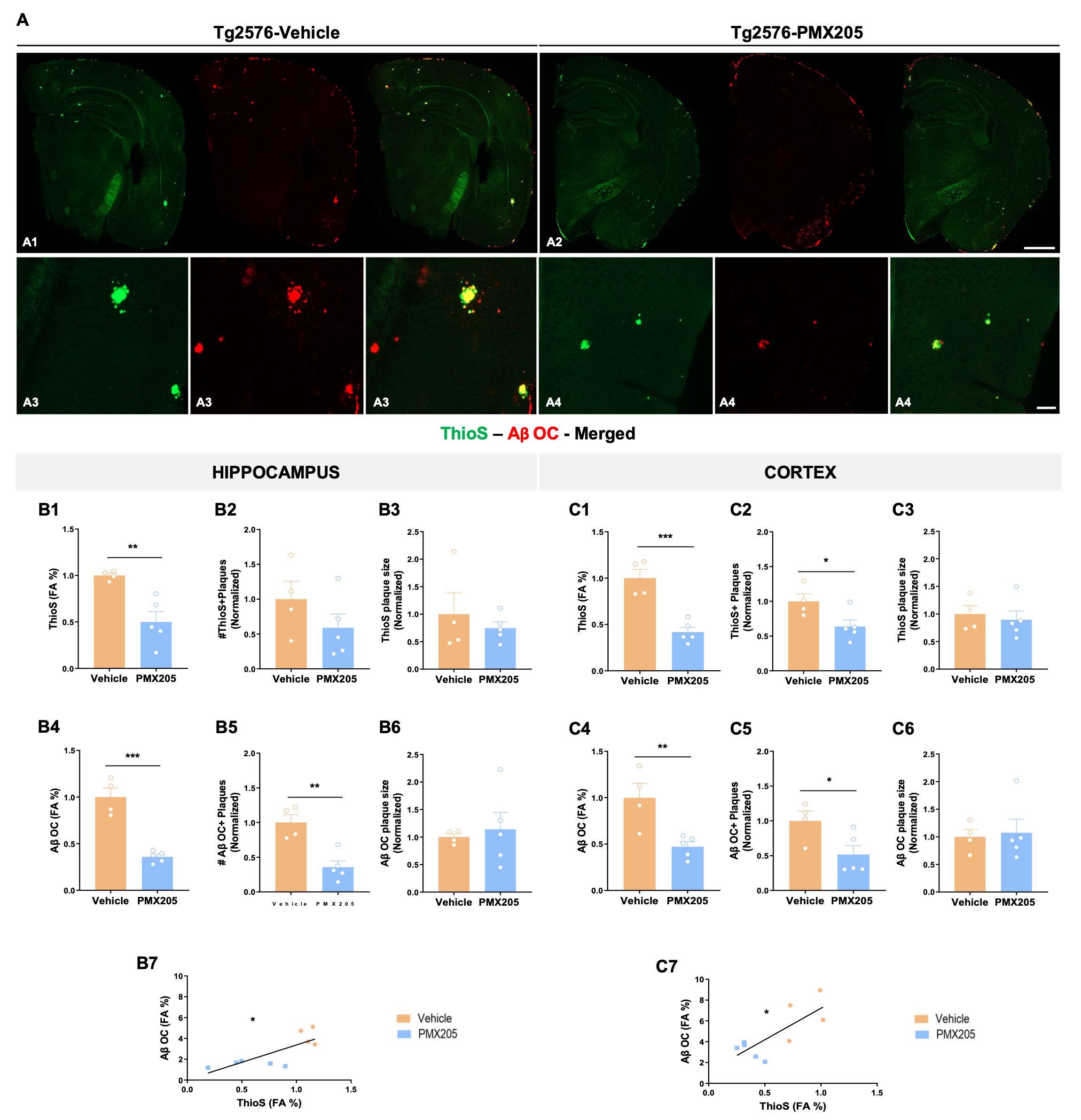

Supplement: Supplementary file 2 — Additional file 2: Figure S2. Amyloid pathology decrease with PMX205 in the Tg2576 mouse model of Alzheimer’s disease. (A) Representative stitched images of brain hemispheres of Tg2576-vehicle (A1) and Tg2576-PMX205 (A2) stained with ThioS, and OC. Inserts (A3-A4) shows higher magnification images of Aß deposition in the cortex. (B-C) Quantification of ThioS field area percent (FA%) showed a significant reduction of amyloid burden in the hippocampus (B1) and the cortex (C1). Similarly, Aβ-OC FA% is strongly reduced in both regions analyzed (B4 and C4). Quantification of ThioS+ and OC+ number of plaques (B2, C2, B5 and C5) and size of plaques (B3, C3, B6 and C6) revealed a reduction in the total number but not in the size of amyloid deposits. Positive correlation of ThioS FA% and Aβ-OC FA% in both, hippocampus (R2 = 0.6), and cortex (R2 = 0.58) (B7 and C7). Data are shown as Mean ± SEM and normalized to control group (Tg2576-vehicle). Statistical analysis used a two-tailed t-test and Pearson’s correlation test. Significance is indicated as * p < 0.05; ** p < 0.01 and *** p < 0.001. 3 sections/mouse and n = 4-5 mice per group. Scale bar: A1-A2: 1000 µm; A3-A4: 100 µm. [file 40478_2022_1416_MOESM2_ESM.tif]

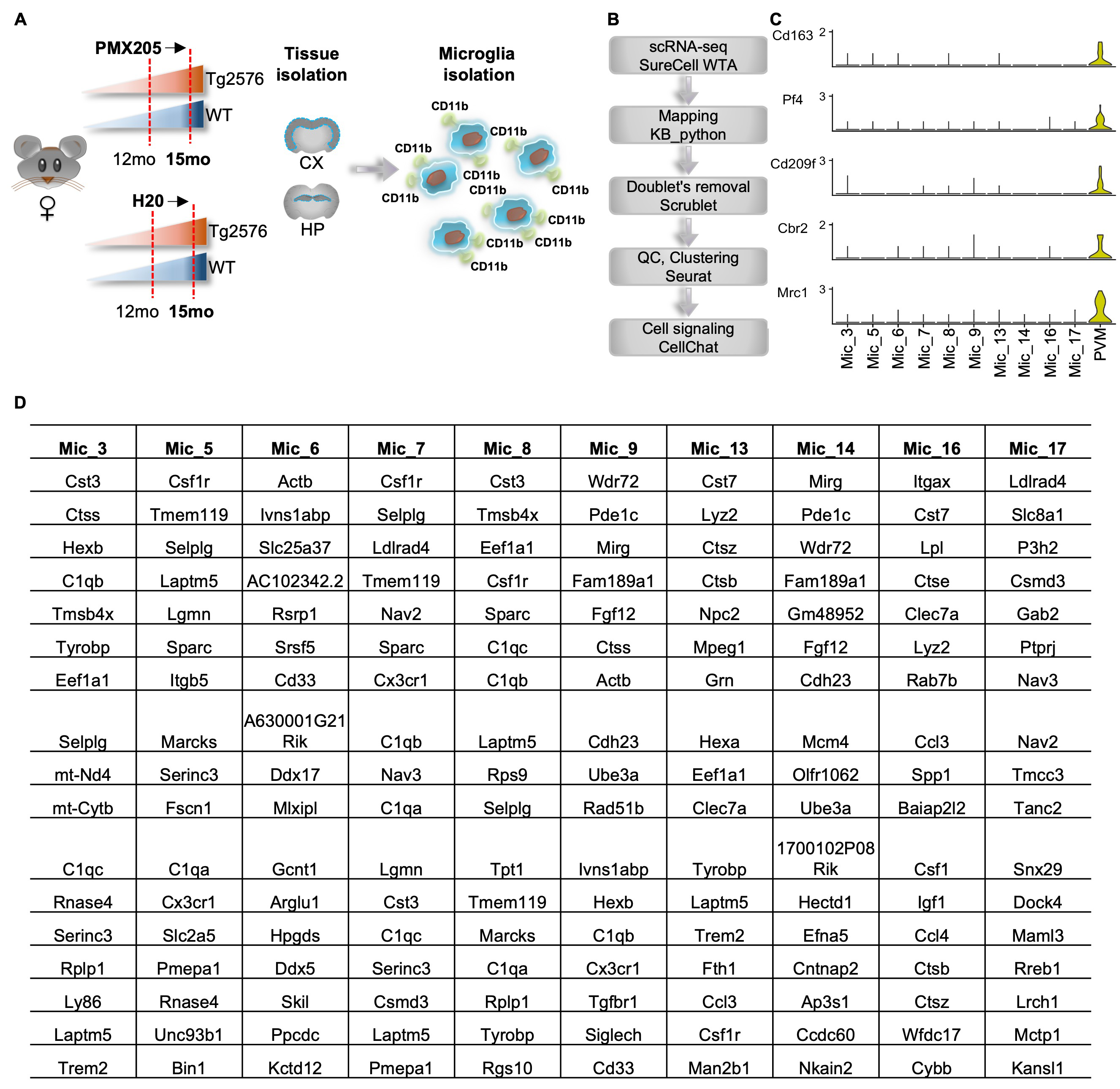

Supplement: Supplementary file 3 — Additional file 3: Supplemental Figure S3. Single-cell RNA-seq of WT and Tg2576 female mice reveals distinct populations of macrophage and microglia (A) Single-cell RNA-seq data collection pipeline. (B) Single-cell RNA-seq data analysis pipeline. (C) Expression of canonical perivascular macrophage markers across subpopulations. (D) Top 17 markers per microglial cluster (logFC >0.25, positive only). [file 40478_2022_1416_MOESM3_ESM.tif]
